# Distinguished University Professor Vladimir Hachinski: Adapted Interview from the 12^th^ World Congress for NeuroRehabilitation (WCNR), Vienna, 2022

**DOI:** 10.25122/jml-2023-1003

**Published:** 2023-01

**Authors:** Stefana-Andrada Dobran, Alexandra Gherman

**Affiliations:** 1 RoNeuro Institute for Neurological Research and Diagnostic, Cluj-Napoca, Romania; 2 Sociology Department, Babes-Bolyai University, Cluj-Napoca, Romania

**Interviewer: Ștefana - Andrada Dobran**

**Interviewee: Professor Vladimir Hachinski**

Department of Clinical Neurological Sciences

Department of Epidemiology and Biostatistics

Robarts Research Institute

Schulich School of Medicine & Dentistry

Western University, London, Ontario, Canada

Vladimir Hachinski, CM, MD (Toronto), DSc (London), FRCPC, FRSC, four times Doctor Honoris Causa, is a Professor of Neurology and Epidemiology and a Distinguished University Professor, Scientist at Robarts Research Institute, University of Western Ontario, Canada. He has made major contributions to the understanding, diagnosis, treatment, and prevention of stroke and dementia. He has published 17 books and over 1,000 articles. He was President of the World Federation of Neurology. He introduced the concept of vascular cognitive impairment – the vascular treatable and a preventable component of most dementias and devised a method of identifying it (an ischemic score). In 2017, he received the Prince Mahidol Award in Public Health, and in 2022 he received the Potamkin Prize, which is considered the "Nobel Prize" for achievements in the field of dementia. He leads a Dementia Prevention/Brain Health Initiative.

**S.A.D.:** Dear Professor Hachinski, we are here, in Vienna, for the 12^th^ World Congress for Neurorehabilitation [organised] by the WFNR. What is your first-hand opinion of the event so far, and have you participated in any previous editions?

**V.H.:** I have never been to a world congress, but I've been to others, like the European ones. Well, the first impression [is] the richness of the things that are presented. There's a lot of information and good information. And the quality of the speakers is quite amazing. [It is] well organised. Now, I think that if there is a theme, it is "multidisciplinary". However, I think that the next step is integration. I wondered whether it would be possible to take some of the different subjects in an area and then offer mini-qualifications. In other words, a certificate, some sort of evidence that a person has acquired the skills necessary for a particular area. And I would emphasize the teaching by using different cases so that when you see a patient with, let's say, Parkinson's disease, there might be two or three typical presentations, then you can relate your patient to the guidelines for managing such a case. So, I think the remarkable thing is the connection with other specialities, particularly neurological specialities, but also technology and, of course, the latest basic science and the potential of brain plasticity and rehabilitation.

**S.A.D.:** What do you believe is the overarching theme of this year's congress?

**V.H.:** Multidisciplinarity. I think that's the overarching theme because there's such a variety of topics. I think the only shortcoming is that there's so much to offer that sometimes you want to go to two different sessions that are at the same time, and you cannot. So, in some ways, I think consolidation would be helpful because the organisation is superb. Clearly, the organisers have the capability of inviting top people, and I look forward to the meeting in Vancouver; it will be even better.

**S.A.D.:** From your perspective, what is the role of hybrid multidisciplinary events in developing neurorehabilitation research and practice, and what other avenues do you believe are worth exploring?

**V.H.:** Well, first of all, I think "hybrid" is the future. There have been a few good things about the pandemic, and that is the tremendous increase in connectivity. Access to patients, access between different specialities, and also the integration of many efforts. And the ability to be looking after some patients, some of the time at their homes. And so, I think it's very important to establish a personal relationship. It's never the same to zoom or to see someone on video as compared to seeing them in person. But once that rapport is established, it is helpful to actually visit them in their home by video and look into their environment and be able to have other members of that family participate in the rehabilitation. Because the resources are limited and we have to call up not only the professionals, but also volunteers and caregivers to make sure that we provide the best we can with the resources that we have.

**S.A.D.:** Could you please tell us a bit about your recent project, "*A comprehensive customised cost-effective approach to the prevention of dementia*" (CCCAP)?

**V.H.:** Yes. There is no comparison between the effectiveness of prevention and acute care and even rehabilitation. Prevention is more effective. I began my career with acute stroke and became very proud of how well we can treat stroke patients. But we can look at it in another way: every stroke is a prevention failure. And we can do much better. My theme is – this afternoon, "*Can we do better in preventing dementia, stroke and heart disease?* " They share the same treatable risk factors. The main one is hypertension. Hypertension is highly prevalent. The chances of an individual developing high blood pressure are 80% in a lifetime. Only half the people who have high blood pressure know that they have it, and among those who know that they have it, only half have it under control. So, it is, on the one hand, a tremendous problem; on the other hand, [it is] an immense opportunity, because we know how to treat hypertension. The first two steps are exercise and diet, and especially cutting out salt, sugar, and trans fats. And then the next step is drugs. And there are many effective, cheap drugs that are widely available across the world. […] So, when I see a patient, I ask whoever is coming with the patient – a spouse or often a daughter - and I ask them two questions: "Do you know the symptoms of a stroke?" Most people don't. Everybody needs to know, even children, because if there is somebody you know, who has sudden loss of speech and they cannot move one side or they lost balance, they need to get an ambulance or let somebody know. So, everybody needs to know. So, if they don't know, I teach them. And the next time they visit, I ask them if they remember. And the second question I ask is – "Do you know your blood pressure?" If they don't know their blood pressure, then I usually arrange for the office to have it taken right there. And if the blood pressure is high, I say, "You know, you'd better follow up on this, you need to treat this". So, these are two very simple things that make a big difference. Because unless people know the symptoms of what a stroke is, they may end up having one. The symptoms may disappear, but the risk doesn't. So, that's so simple and yet so important. And, as you probably know, people having a stroke have double chances of developing dementia. So, one way of preventing some dementia is [to] prevent stroke.

**S.A.D.:** How can multidisciplinary approaches and international collaborative efforts help reduce the burden of stroke on patients?

**V.H.:** The question is how can the different disciplines collaborate. I think the answer is that they should identify what they have as a common interest. For example – I'm Canadian, so I'll talk about the Canadian situation, but there are equivalent situations in most countries of the world. In Canada, we have The Heart and Stroke Foundation; we have The Alzheimer Society, we have Hypertension Canada. And so, each is interested in prevention. But they don't do it together. High blood pressure is a risk factor for stroke, for heart disease, and for dementia. So, why not get together in campaigns to control blood pressure [so] that you are preventing all three?! It's cheaper, [and] you get more people involved. And the other thing that's happening is, of course, [that] there are many more wearables. I mean, these are little things that people can wear, and so, for example, sleep can now be monitored by having a little device. You can monitor the heart if you have atrial fibrillation, which is an irregularity that can lead to stroke and also dementia. So, to go back to your previous question, we have to involve everybody in brain health and also, what I am saying, [is that] not only do you have to do this because 20 years from now you can have dementia or stroke, you have to do it now because you'll feel better. If you exercise, if you eat well, if you sleep well, you'll feel better, and you'll perform better.

**S.A.D.:** And how can multidisciplinary approaches reduce the burden of stroke on the healthcare systems?

**V.H.:** Well, I think if there are fewer strokes, there are fewer dementias; it's easier for everybody. And those resources can be used in education. In the digital age and the knowledge-based economy, everybody needs to learn all the time. For example, with the pandemic – I don't like computers - however, I had to learn a lot. I learned how to Zoom, how to lecture virtually, and how to do other things. And so, this will continue to change. And I have no choice but to continue learning. And, certainly, young people will not have one profession. I've been a doctor all my life, but nowadays, people have one, two, three, professions because demands for skills change. And so, I see universal education for everybody, all the time. Things have become easier: you can combine "in person", which is very important, with virtual. So, I think "hybrid" is part of the future and is going to increase.

**S.A.D.:** What are the most at-hand instruments to increase awareness of the impact of stroke and dementia?

**V.H.:** The most important thing is to reach people in what I am calling "*an actionable community*". We, doctors, are not very good at asking for the help of sociologists and demographers who study populations and how people organise themselves. It turns out that if you want to do something and be very effective at it, the number to be involved is about 7, gives or take 3. If it's larger than that, people don't feel they have that sense of commitment. On the other hand, people have networks. You have friends who influence each other. For example, in the United States, they did a study whereby they found that if you are obese, your friends have a 50% chance of also being obese. And your friends' friends have a 20% chance of being obese. So, obesity can spread. These are bad things. But, on the other hand, if I quit smoking, then there is a 50% chance that my friend will stop smoking and a 20% chance that their friends will stop smoking. So, we have a lot to learn [about] how people get organised and how we get take advantage of some of these human characteristics. I'm optimistic because it turns out that it doesn't take that many people to change a habit. For example, when I was growing up, everybody was smoking, particularly women; it was cool. Now you look at her [and say] "Oh, my God! That woman smokes? She stinks!" It's a changing culture. So, we have to change how we look at things. We want to be healthy, exercise, and sleep well, and that becomes the norm. And they get a few people who can champion that. We have examples of athletes - for example, soccer players have terrific brains to decide to kick a ball in a split second. We tend to think of "brainy"; we think of professors. But everybody needs a brain, so people who do sports, who build things, and people who drive race cars, have wonderful brains. So, I think this is the most valuable thing. The brain represents the three most precious pounds in the whole universe. If the brain were an expensive car that would last a lifetime, we would treat it better than we treat our brains.

**S.A.D.:** Definitely.

**V.H.:** So, we should treat a brain as well as a very expensive, exclusive, one-in-a-lifetime car.

**S.A.D.:** Thank you very much, Professor! Thank you for being here!

**V.H.:** It was my pleasure to be here, and thank you for interviewing me!

